# Supplementary material for: Magnetic Fe/Fe3C@C Nanoadsorbents for Efficient Cr (VI) Removal
Source: Int J Mol Sci. 2022 Dec 1;23(23):15135. doi: 10.3390/ijms232315135 (PMC9739629; doi:10.3390/ijms232315135)
Supplement: Supplementary file 1 [file ijms-23-15135-s001.zip › ijms-2006779-supplementary.pdf]

International Journal of
Molecular Sciences

MDPI

Supplementary materials

Magnetic carbon nanocomposites from Fe3O4 reduction and their application as Cr (VI) adsorbents

Laura Cervera-Gabalda [1,2] and Cristina Gómez-Polo [1,2,*]

[1] Departamento de Ciencias, Universidad Pública de Navarra, Campus de Arrosadia, 31006 Pamplona, Spain
[2] Institute for Advanced Materials and Mathematics (INAMAT2), Universidad Pública de Navarra, Campus de Arrosadia, 31006, Pamplona, Spain
[*] Correspondence: gpolo@unavarra.es; Tel.: +34-948169576

Supplementary Figures

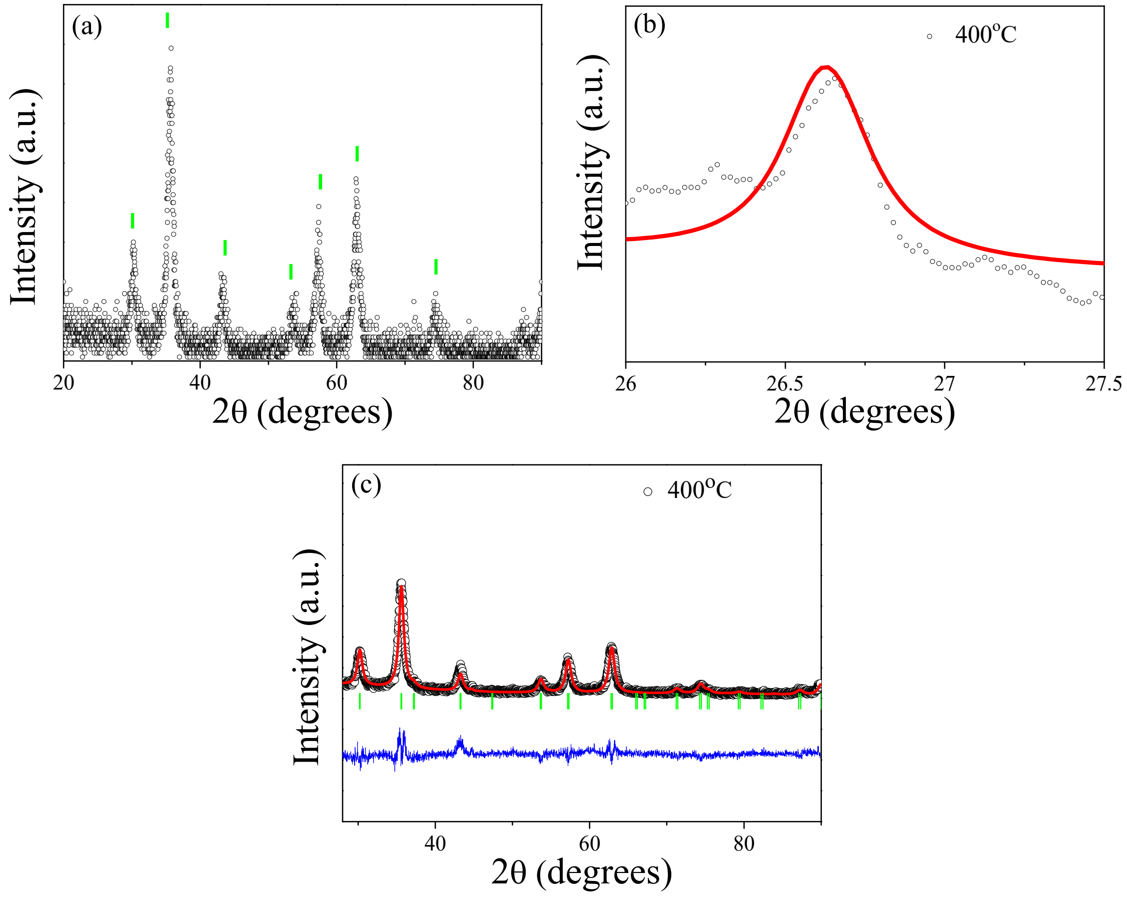

Figure S1. XRD patterns for the (**a**) Fe_3O_4 initial MNPs and (**b**,**c**) MNPs + fructose sample at T_{ann} = 400°C: (o) Experimental, (—) calculated (Rietveld refinement) intensities and (—) difference between both intensities. The Bragg reflections are shown for (|) Fe_3O_4.

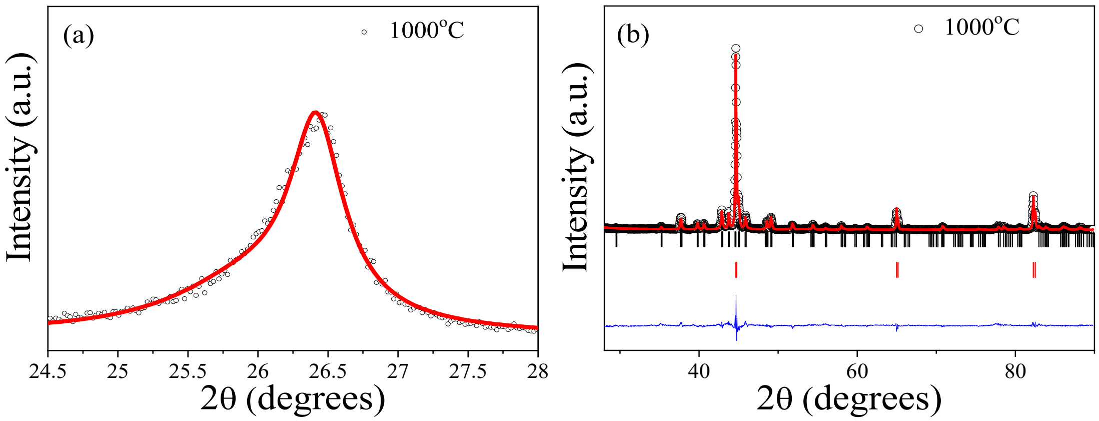

Figure S2. XRD patterns for the MNPs + fructose sample annealed at T_{ann} = 1000ºC. (o) Experimental, (—) calculated (Rietveld refinement) intensities and (—) difference between both intensities. The Bragg reflections are shown for (|) Fe_3C and (|) α-Fe.

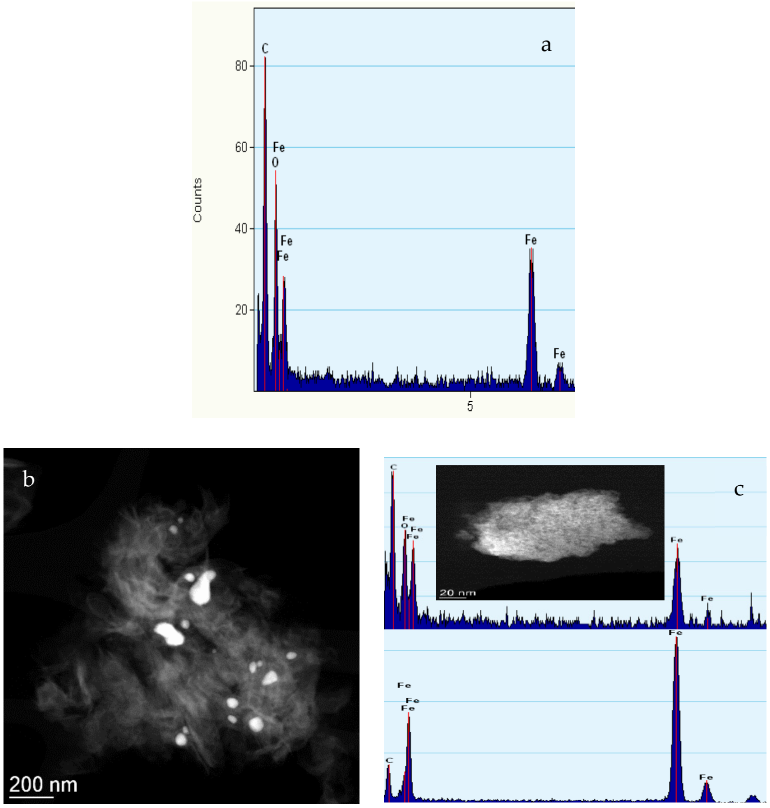

Figure S3. (**a**,**c**) EDX analysis for the MNPs + fructose sample annealed at T_{ann} = 400 and 600°C, respectively. (b) STEM image of the MNPs + fructose sample annealed at T_{ann} = 600°C.

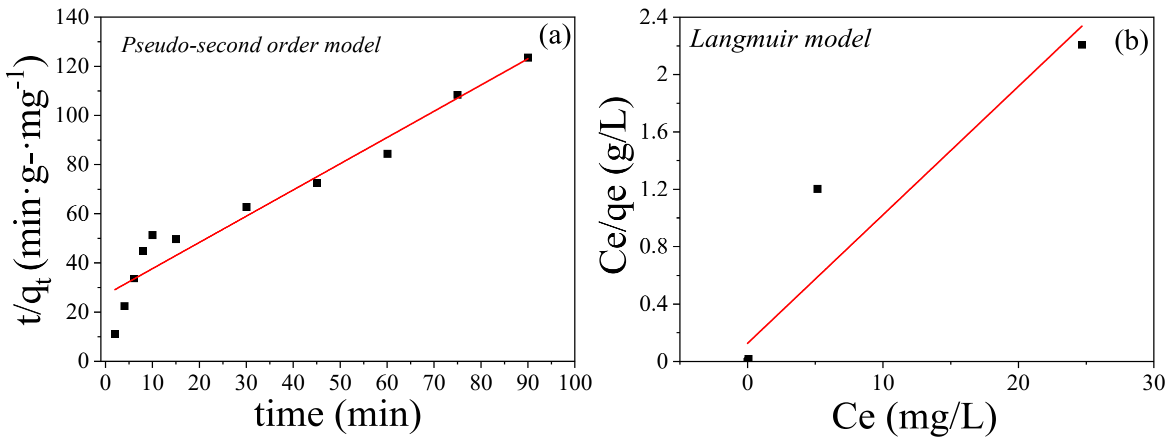

Figure S4. (a) Adsorption kinetics of Cr(VI) and (b) adsorption isotherms in the presence of the initial Fe_3O_4 MNPs.
